# Radiological manifestations of thoracic hydatid cysts: pulmonary and extrapulmonary findings

**DOI:** 10.1186/s13244-020-00916-0

**Published:** 2020-11-11

**Authors:** Gamze Durhan, Aziz Anıl Tan, Selin Ardalı Düzgün, Selçuk Akkaya, Orhan Macit Arıyürek

**Affiliations:** 1 Department of Radiology, Hacettepe University Faculty of Medicine, 06410 Ankara, Turkey; 2 Department of Radiology, Karadeniz Technical University Faculty of Medicine, Trabzon, Turkey

**Keywords:** Thoracic hydatid cyst, Pulmonary, Extrapulmonary

## Abstract

Hydatid cyst caused by the larval form of *Echinococcus* is a worldwide zoonosis. The lungs and liver are the most common sites involved. While the lung parenchyma is the most common site within the thorax, it may develop in any extrapulmonary region including the pleural cavity, fissures, mediastinum, heart, vascular structures, chest wall, and diaphragm. Imaging plays a pivotal role not only in the diagnosis of hydatid cyst, but also in the visualization of the extent of involvement and complications. The aim of this pictorial review was to comprehensively describe the imaging findings of thoracic hydatid cyst including pulmonary and very unusual extrapulmonary involvements. An outline is also given for the findings of complications and differential diagnosis of thoracic hydatid cyst.

## Key Points

- Imaging plays a crucial role in the diagnosis of hydatid cyst in common pulmonary and uncommon extrapulmonary locations.
- Uncomplicated pulmonary hydatid cysts are seen on computed tomography as well-defined homogeneous lesions with low density and smooth walls of variable thickness.
- Pulmonary rupture of hydatid cyst can be classified as contained or complete rupture.
- While imaging findings of contained rupture are listed as air crescent sign, inverse crescent sign, and air bubble sign, imaging features of complete rupture are cumbo sign, whirl sign, waterlily sign, rising sun sign, mass within the cavity sign, and dry cyst sign.
- Unusual locations of extrapulmonary hydatid cysts are the chest wall (bones and soft tissues), pleural cavity and fissures, diaphragm, mediastinum, heart chambers, and vascular structures including both arteries and veins.

## Introduction

Hydatid cyst (HC) caused by the larval form of *Echinococcus* is a worldwide zoonosis, which is most commonly caused by *Echinococcus granulosus* (EG). The other less common but more aggressive type is caused by *Echinococcus multilocularis* [1, 2]. Although the liver is the most common site for HC, it can involve almost any organ of the body except hair, teeth, and fingernails. The lungs are the second most frequent location of hematogenous spread in adults and probably the most common location in children [3, 4]. While the lung parenchyma is the most common site within the thorax, it may develop in any extrapulmonary region including the pleural cavity, fissures, mediastinum, heart, vascular structures, chest wall, and diaphragm. Imaging plays a pivotal role not only in the diagnosis of HC, but also in the visualization of the extent of involvement and complications [2, 5]. Although the classical imaging findings of liver HC are well known, the findings of thoracic HC, especially the involvement of extrapulmonary locations, have been less frequently described in the literature.

**Fig. 1:**
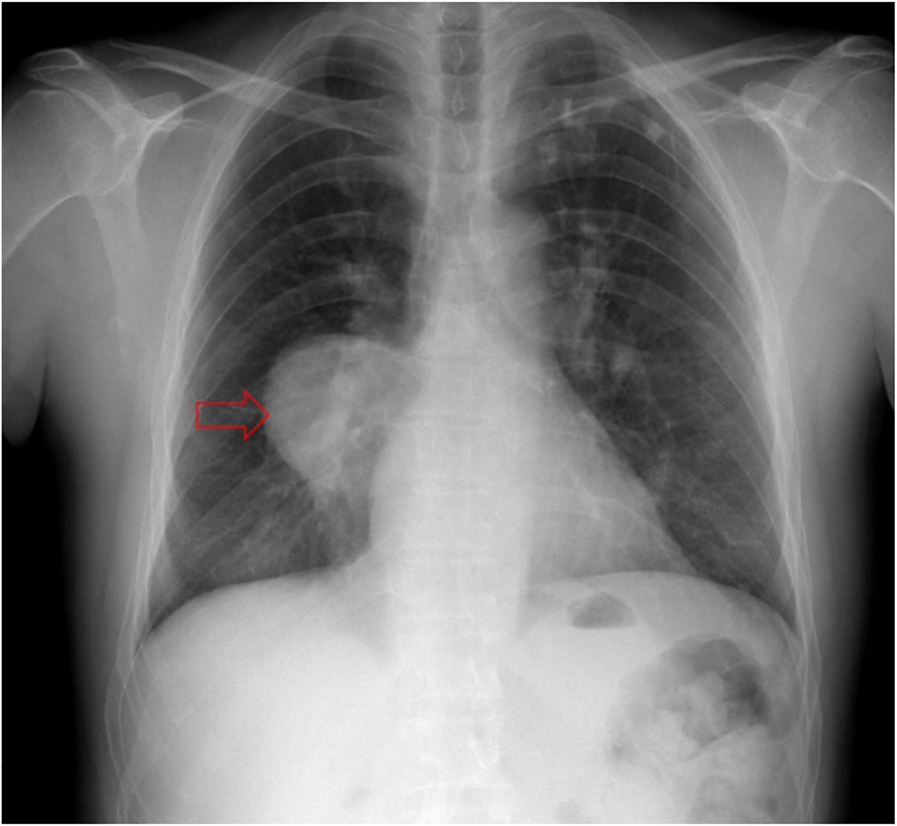
Hydatid cyst has a non-specific appearance on chest radiography

Plain chest radiography, ultrasonography, computed tomography (CT), and magnetic resonance imaging (MRI) may show HC. Although chest radiography is the primary diagnostic method because of its common usage, it is inadequate for the assessment of complications and spread (Fig. 1). However, ultrasonography can show HCs located on the diaphragm and periphery of the lung while transthoracic echocardiography can demonstrate HCs located in the heart chambers. Nevertheless, both ultrasonography and transthoracic echocardiography are not sufficient to show HC extensions. CT and MRI can demonstrate features, complications, and extension of HCs in detail. In addition to providing diagnosis and showing complications of HCs, CT and MRI can play a role in evaluating treatment efficacy by demonstrating changes in the size, number, and shape of HCs. Therefore, the imaging findings of thoracic HC described in this paper are based on CT and MRI.

Although non-contrast thoracic CT can be obtained to evaluate pulmonary HCs, a contrast agent is necessary to demonstrate extrapulmonary HCs, especially those located in the mediastinal structures. Patients were imaged in a supine position, with the arms extended overhead and instructed to hold their breath during the acquisition. After injecting an iodinated contrast agent into the antecubital vein via a 21-gauge catheter using a dual-chamber power injector, 30 mL of saline was infused. Venous phase imaging was performed with a 40–60-s delay. Craniocaudal acquisition was acquired from the apex to the upper abdomen. The CT parameters were modulated mAs, with reference 100 mAs, 120 kVp, rotation time 0.25 s, and collimation of 192 × 0.6 mm with a pitch of 2.5.

For chest MRI, patients were imaged in a supine position with the head pointing towards the magnet. Axial, coronal, and sagittal T2-weighted, axial diffusion-weighted, precontrast axial T1-weighted sequences with and without fat suppression; T1-weighted sequence in- and out-of-phase; and postcontrast T1-weighted sequence with fat suppression were obtained.

The aim of this pictorial review was to comprehensively describe the imaging findings of thoracic HC including pulmonary and very unusual extrapulmonary involvements. An outline is also given for the findings of complications and differential diagnosis of thoracic HC.

## Pulmonary involvement

**Fig. 2:**
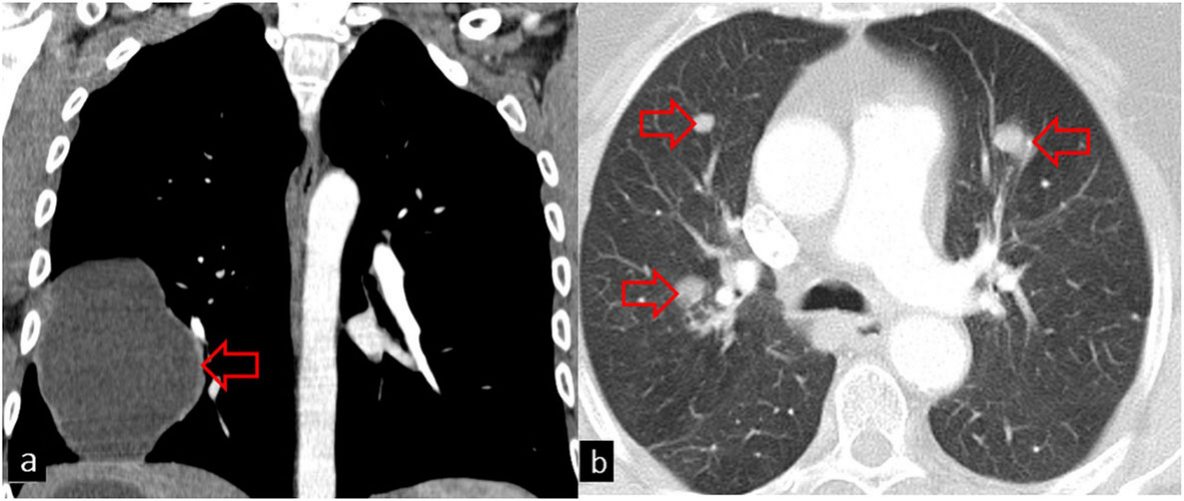
**a** Coronal chest CT image in the mediastinal window shows a giant (> 10 cm) uncomplicated hydatid cyst (red arrow). Measurement of HU values on CT shows low density (mean HU ± SD = − 1 ± 10) consistent with simple fluid. **b** On axial CT image, bilateral hydatid cysts are seen (red arrows)

The pulmonary parenchyma is the second most frequent site of involvement in adults (10–30%) and the most common site of involvement in children and young adults [6, 7]. The lungs facilitate cyst growth because of their compressibility and negative pressure. Therefore, the size of pulmonary parenchymal HC may vary from 1 to 20 cm [8, 9]. Giant HC is usually defined as a cyst with the largest diameter of more than 10 cm and is more commonly reported in children than in adults due to the immature immune system and greater elasticity of the lung tissue. Pulmonary HCs are mainly located in the lower lobes (55–70% of cases) and maybe multiple (30%) and bilateral (20%) [2, 3, 7, 8, 10–12] (Fig. 2). Pulmonary HCs usually remain asymptomatic until they rupture. Clinical symptoms such as sudden coughing attacks, hemoptysis, dyspnea, fever, and chest pain can be seen. Although allergic reactions may develop due to cyst rupture, fatal anaphylaxis is uncommon [2, 13].

**Fig. 3:**
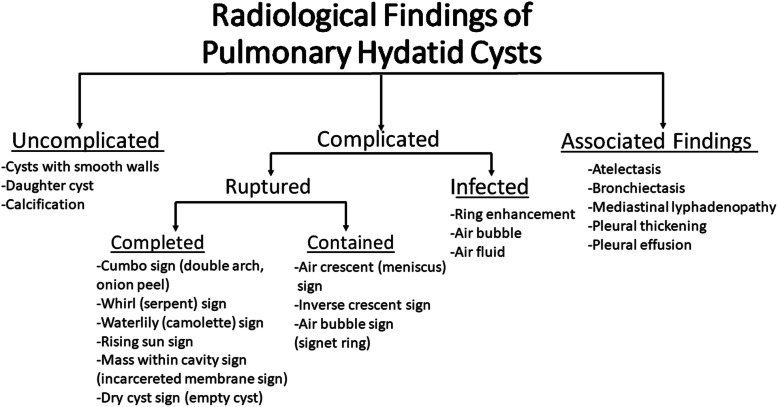
Radiological findings of pulmonary hydatid cysts

Radiological manifestations of pulmonary HCs can be variable depending on the presence of complications. They can be classified as uncomplicated and complicated (contained rupture, complete rupture, superinfection). Besides pulmonary HCs, associated thoracic findings can be seen (Fig. 3).

**Fig. 4:**
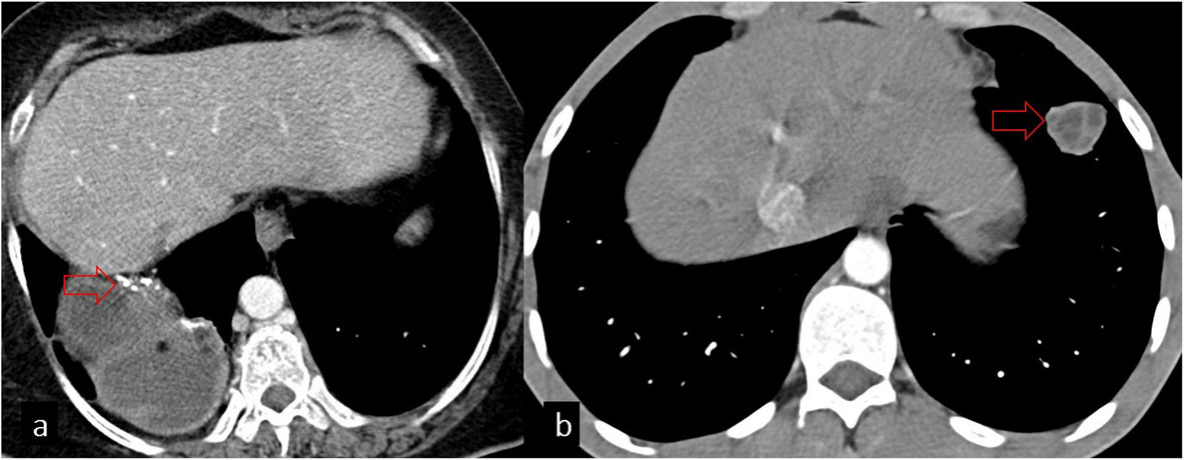
**a** Calcification in the wall of the hydatid cyst is seen on the axial CT image (red arrow). **b** Daughter cysts in the hydatid cyst (red arrow)

*Uncomplicated* pulmonary HCs are seen as well-defined homogeneous lesions with smooth walls of variable thickness. Density measurements on CT show low HU values consistent with fluid content. While centrally located cysts are usually round, peripheral cysts may be oval or polycyclic [2, 7]. Daughter cysts and calcifications are rarely seen in pulmonary HC [7, 13] (Fig. 4).

*Complicated* cyst is defined as a ruptured and/or infected cyst. Rupture of pulmonary HC can occur in up to 47.5% of cases. Several factors including age, antihelminthic therapy, chemical reactions, size of the cyst, and immune system of the host can cause rupture via degeneration of cyst membranes [7, 8, 10, 14]. Unlike uncomplicated HCs, complicated HCs may show higher HU values due to mucus, infection, or hemorrhagic content.

*Rupture* can be classified as contained or complete rupture.

**Fig. 5:**
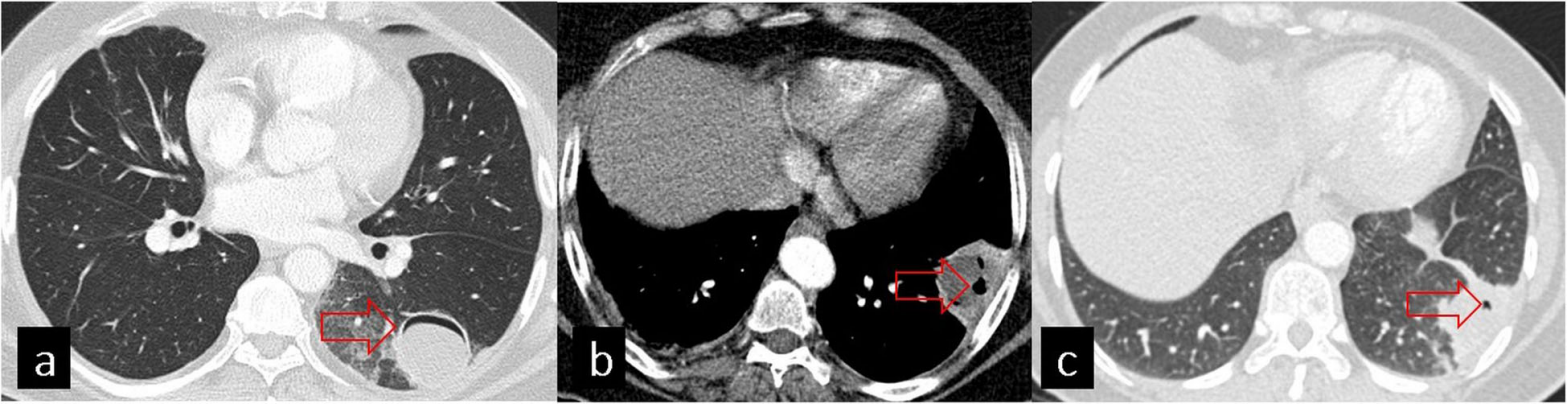
Imaging features of contained rupture. Axial CT images show the crescent sign (**a**, red arrow), inverse crescent sign (**b**, red arrow), and air bubble sign (**c**, red arrow)

*Contained rupture* is described as a detachment of the pericyst from the endocyst. As the cyst contents are surrounded by the pericyst, a contained rupture is unlikely to develop as an allergic reaction or infection. Imaging features of contained rupture can be listed as follows: air crescent sign, inverse crescent sign, and air bubble sign (Fig. 5).

*Air crescent sign (meniscus sign)*: a thin crescent of air is seen between the pericyst and endocyst caused by bronchial erosion.

*Inverse crescent sign*: an air crescent is seen at the posterior aspect of a lesion created by dissection through the posterior side of membranes.

*Air bubble sign (signet ring sign)*: small intracystic air blebs are seen at the periphery of the cyst between the pericyst and endocyst [2, 8, 10, 11, 15].

**Fig. 6:**
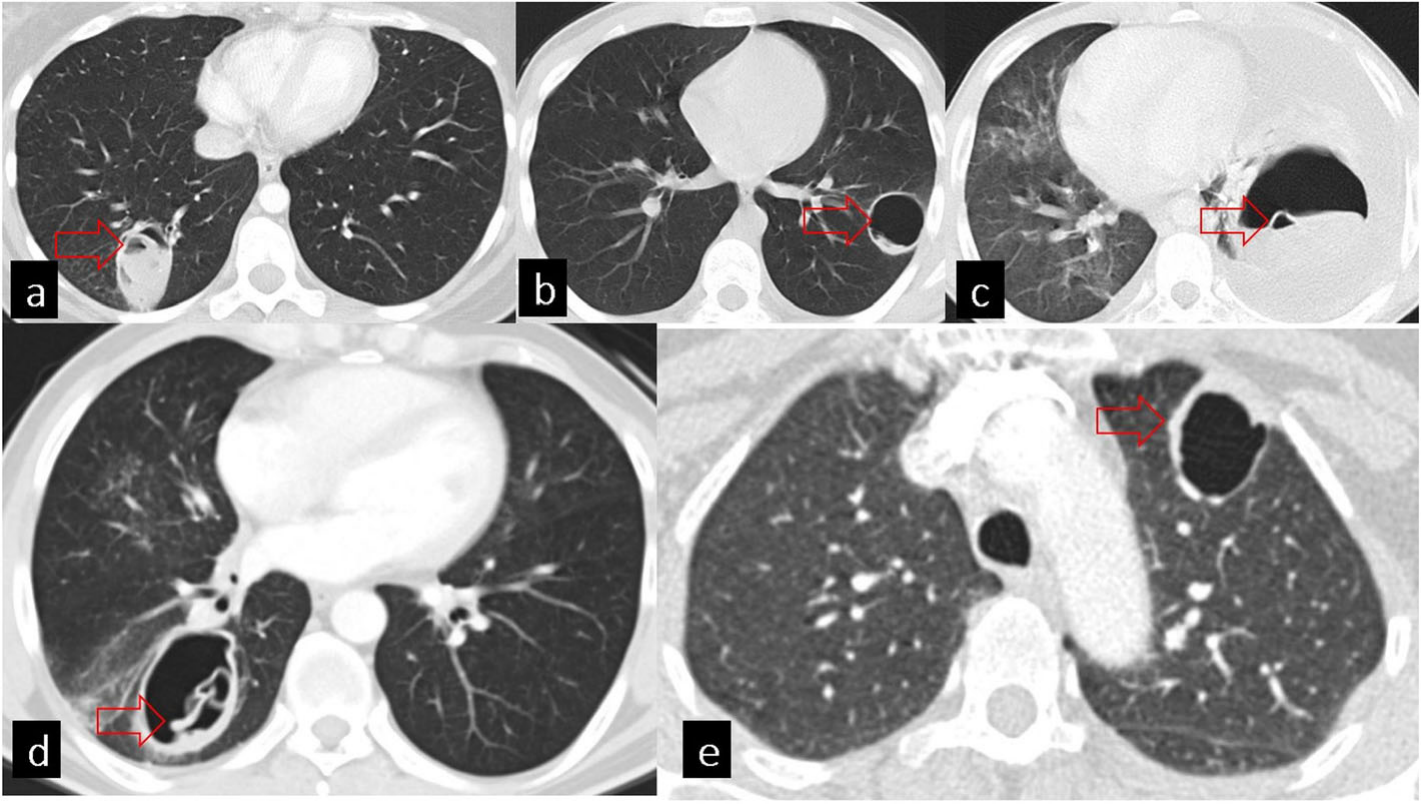
Complete rupture signs. Axial CT images of different patients on the lung window (**a**–**e**). **a** Cumbo sign (red arrow). **b** Mass within cavity sign (red arrow). **c** Waterlily sign (red arrow). **d** Whirl sign (red arrow) **e.** Empty cyst sign (red arrow)

*Complete rupture* is defined as the presence of signs of connection with the bronchus. Radiological findings of complete rupture can be listed as follows: cumbo sign, whirl sign, waterlily sign, rising sun sign, mass within the cavity sign, and dry cyst sign (Fig. 6).

*Cumbo sign (double arch sign, onion peel)*: at this stage, the endocyst shrinks and ruptures due to the increasing amount of air. The air-fluid level is seen in the endocyst and the pericyst.

*Whirl sign (serpent sign)*: collapsed membranes within the cyst after expectoration of cyst fluid.

*Waterlily sign (camolette sign)*: with the complete collapse of the endocyst, wrinkled floating membranes are seen in the remaining fluid.

*Rising sun sign*: with the rupture of the endocyst, daughter cysts may appear as round radio-opacities at the bottom of the cysts.

*Mass within cavity sign (incarcerated membrane sign)*: when the fluid is completely evacuated by expectoration, the remaining solid components fall to the dependent part of the cavity and create this sign.

*Dry cyst sign (empty cyst sign)*: with the expectoration of cyst contents, the pericyst is seen to be empty and air-filled [2, 8, 10, 11, 15, 16].

**Fig. 7:**
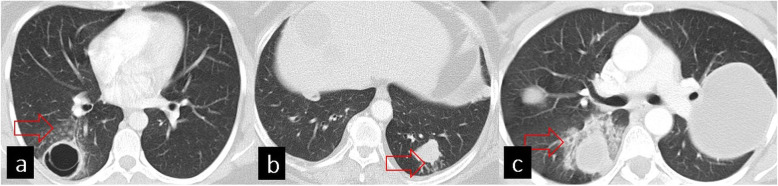
Imaging features of hydatid cyst rupture directly to the lung parenchyma. Axial CT images of different patients showing centrilobular nodular opacities around the hydatic cyst (**a**, red arrow), tree-in-bud pattern around the HC (**b**, red arrow), and consolidation around the HC (**c**, red arrow)

In addition to the rupture of the endocyst, HC can also rupture directly to the parenchyma, main bronchus, and pleural cavity. Rupture of the cyst into the parenchyma can cause consolidation surrounding the cyst, and centrilobular opacities can be seen because of the endobronchial extensions of HCs (Fig. 7).

Rupture of HCs to the pleural cavity can produce hydrothorax or hydropneumothorax. HC also can communicate directly with the bronchus.

*Superinfection* is the most common complication of a ruptured HC [8]. HC with superinfection can show higher HU values and cannot be differentiated from pyogenic abscess. Signs of cyst infection are ring enhancement sign, air bubble sign, and air-fluid level.

*Ring enhancement sign*: thickening of the cyst wall with enhancement.

*Air bubble and air-fluid level*: although air bubble and air fluid can be an indicator of cyst rupture, these signs can also be seen in superinfection.

**Fig. 8:**
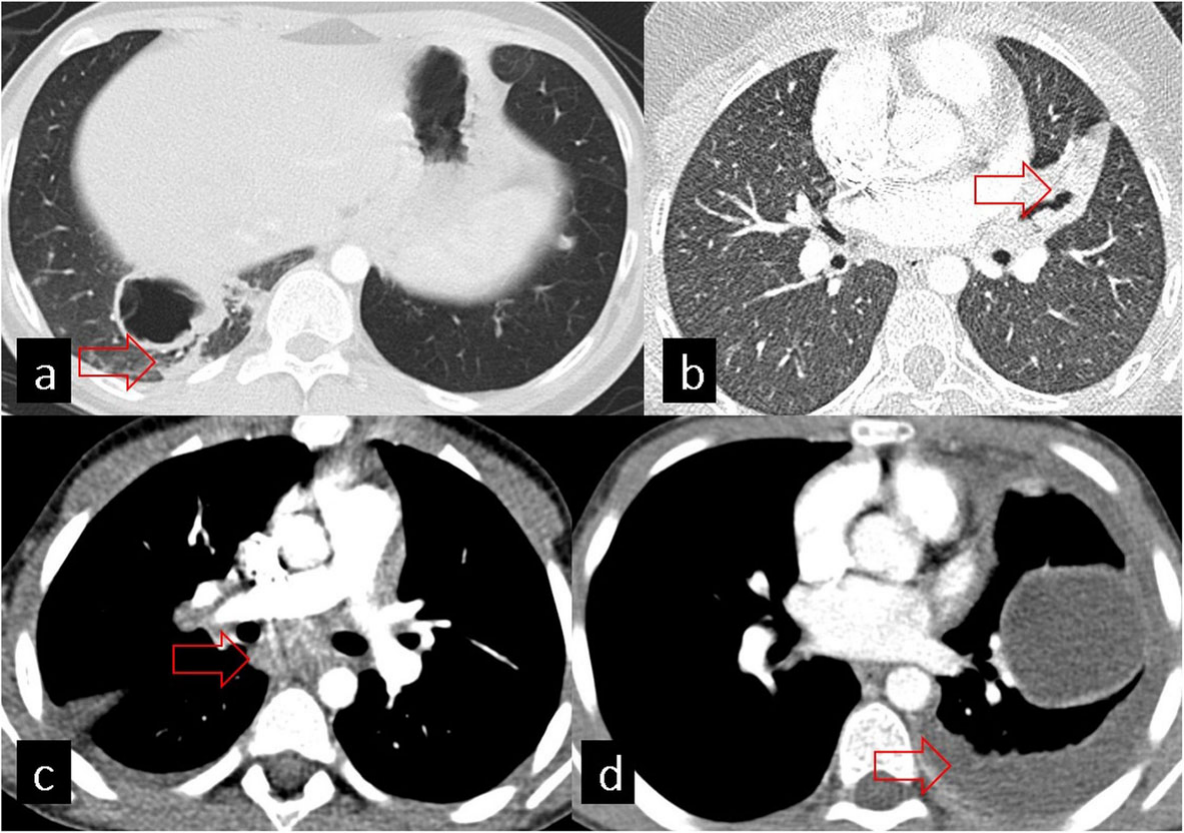
Axial CT images in the lung (**a**, **b**) and mediastinal (**c**, **d**) windows show associated findings of HC. **a** Atelectasis. **b** Bronchiectasis. **c** Mediastinal lymphadenopathy seen especially in hydatic cyst with superinfection. **d** Pleural effusion

In addition to the findings of complicated and uncomplicated HCs, there can be associated thoracic findings including atelectasis, bronchiectasis, mediastinal lymphadenopathy, and pleural thickening or effusion (Fig. 8). Atelectasis can be caused by compression of the HC and may facilitate the development of pneumonia. Bronchiectasis can be seen in the distal parenchyma of HCs due to bronchial obstruction and parenchyma destruction [17]. As another associated finding, pleural thickening or free fluid can develop without intrapleural rupture of the HC [18]. Although rare, mediastinal lymphadenopathy may also be seen, especially in HCs with superinfection [19].

**Fig. 9:**
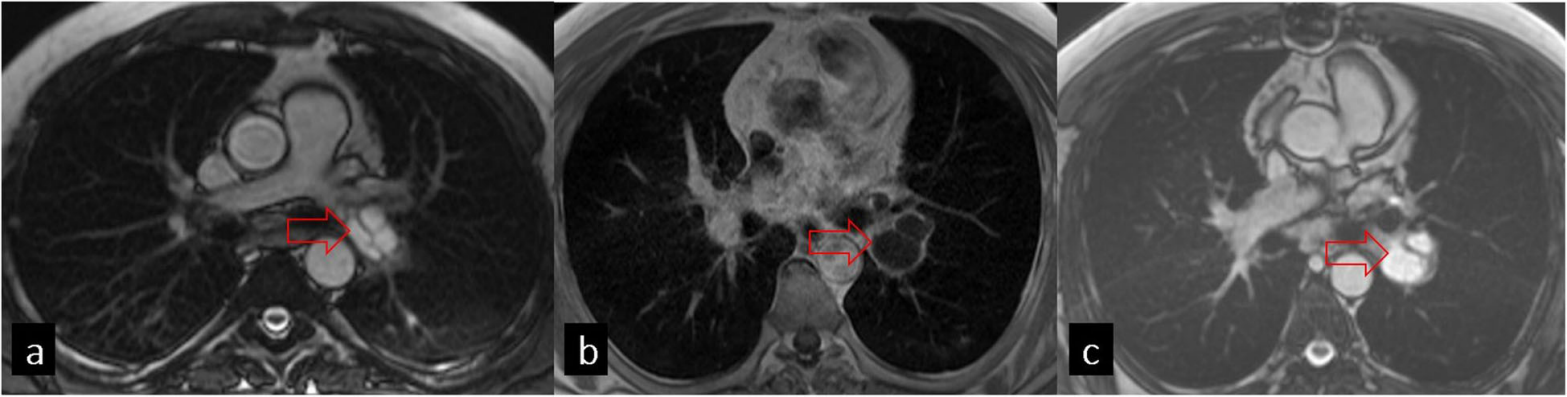
Steady-state free precession (SSFP) gradient echo magnetic resonance image shows a hyperintense hydatid cyst in the left pulmonary artery (**a**), contrast-enhanced T1W image shows hypointensity (**b**), and gradient TRUFI demonstrates hyperintensity in hydatid cysts (**c**)

Although CT is very useful for the diagnosis and showing the extension of HC, MRI is more successful in demonstrating the relationship with adjacent soft tissues and cyst contents, including detached germinal membranes and daughter cysts. HCs have low signal intensity on T1-weighted and high signal intensity on T2-weighted images. The daughter cysts may show low or high signal intensity depending on their varying contents (Fig. 9).

Although more uncommon than EG, pulmonary HCs can also be caused by *Echinococcus multilocularis*, also known as alveolar echinococcus. It is more aggressive than EG and more easily confused with malignancies. Lesions tend to be in the peripheral zones and are seen as low-density masses with lobular contours. They can grow to a larger size and invade adjacent organs more than EG. Microcalcification clusters may be seen, and this finding can help to differentiate it from EG [20].

## Differential diagnosis

**Fig. 10.**
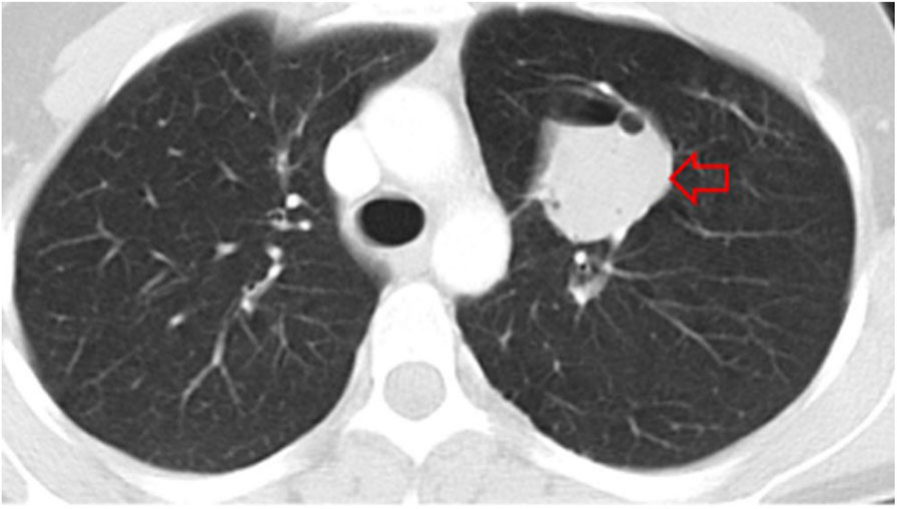
Axial CT image shows an opacification with smooth contours, which is a pathologically proven bronchogenic cyst (red arrow)

Hydatid disease should be always kept in mind in the differential diagnosis when a cystic lesion is detected in a patient who has come from an endemic area. Differential diagnosis for pulmonary HCs can be listed as bronchogenic cyst, lung carcinoma, primary sarcoma of the lung, metastasis (especially for multiple HCs), hematoma, mesothelioma, granuloma, and abscess [2, 21] (Fig. 10).

## Findings of extrapulmonary involvement

Intrathoracic but extrapulmonary HCs are very rare and can occur secondary to hematogenous spread or extension of hepatic, pulmonary, or splenic HCs. Extrapulmonary HCs may develop in the chest wall, pleural cavity, fissures, diaphragm, mediastinum, heart chambers, and vascular structures.

## Chest wall involvement

### Bone involvement

Chest wall involvement of HCs can be in both bony structures and soft tissues. The overall rate of bone involvement of HC has been reported as 0.5–4%. Although the spine is the most common location, HC in the thoracic cage including the ribs and sternum can also be seen [2]. In bone lesions, aggressive proliferation can occur due to the absence of pericyst formation, and the cysts cannot show the typical round shape. Over time, HC replaces the osseous tissue, destroys the cortex, and spreads to adjacent soft tissue. On imaging, bone involvement of HC is seen as well-defined, multiloculated, expansile lytic lesions with irregular branching. While extraosseous HCs may show calcification, intraosseous HCs rarely calcify. The absence of osteoporosis, sclerosis in the involved bone, absence of damage to intervertebral disc spaces, and spread to the paraspinal region are the imaging features of spinal HC. Pure osteolysis sometimes causes a pathological fracture. Therefore, it can present with pain or bulging on the thoracic wall with extension to the surrounding soft tissue.

**Fig. 11:**
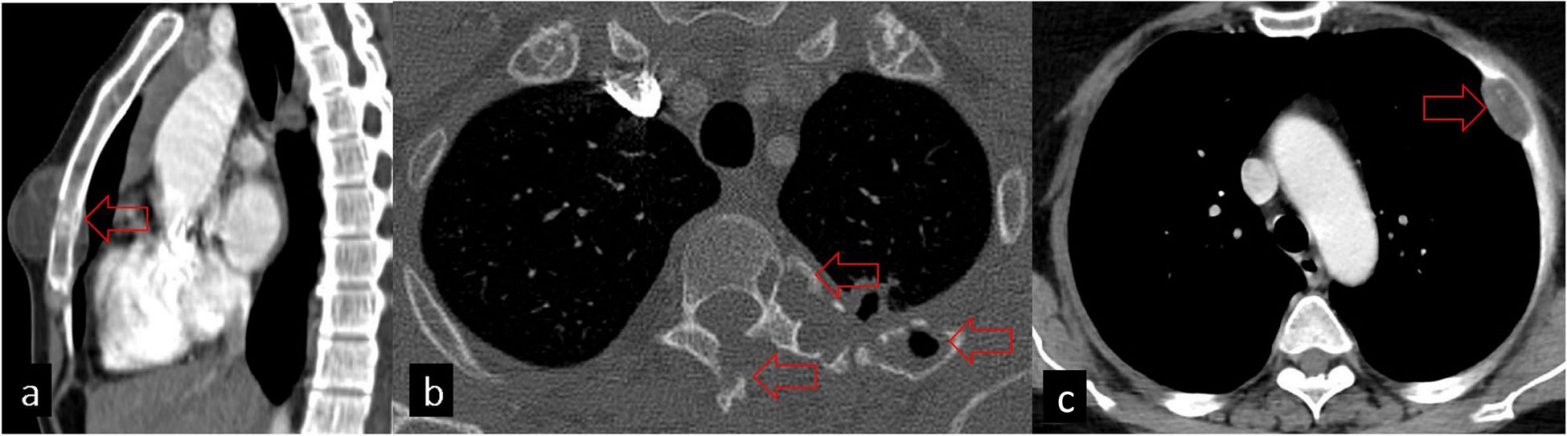
Bone hydatid cysts and differential diagnosis. **a** Axial chest CT image shows an HC located anteriorly adjacent to the sternum causing sternal cortical erosion (red arrow) **b** Axial CT image in the bone window shows the destruction of the vertebra and adjacent costa (red arrows) secondary to HC. **c** Costa destruction secondary to lung cancer is seen (red arrow)

The radiological differential diagnosis of bone HCs includes osteolytic lesions such as metastases, neurofibromas, giant cell tumors, bone cysts, and tuberculosis [2, 7, 22] (Fig. 11).

### Soft tissue involvement

The frequency of primary soft tissue involvement is 0.5–4.7% of cases [23]. Isolated muscular HC is very rare. Muscular involvement is usually seen with extension from other organs. The growth of the HC within a muscle is difficult due to the contractility of the muscles and the presence of lactic acid [7]. In addition to muscular involvement, HC can also be seen in subcutaneous soft tissue, where it may have various appearances including unilocular cyst, multiloculated cyst with daughter cyst, and complex cystic lesion with or without calcification. Multiple HCs can be seen following cyst rupture of the cyst, which can also cause edema and acute inflammation.

**Fig. 12:**
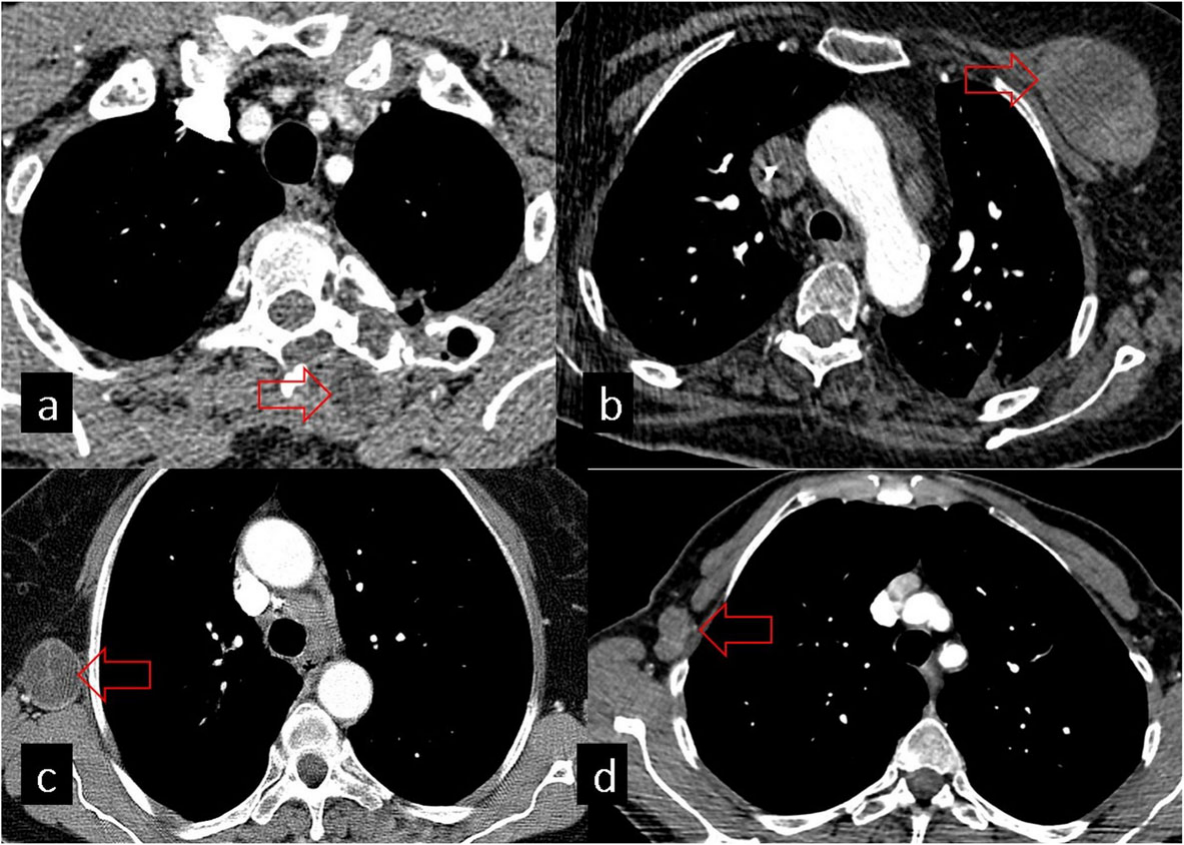
Hydatid cysts in thoracic soft tissues and differential diagnosis. **a** Axial CT image shows left paraspinal muscle involvement of HC (red arrow). **b** Pectoral hematoma as a differential diagnosis of HC seen on CT image (red arrow). **c** HC with daughter cysts located in the right axilla seen on the axial CT image (red arrow). **d** Necrotic lymphadenopathy secondary to lung cancer is seen in the right axilla

The radiological differential diagnosis of soft tissue involvement can be listed as follows: abscess, chronic hematoma, synovial cyst, necrotic soft tissue tumors, lymphangioma, and necrotic lymphadenopathy [7, 24] (Fig. 12).

### Pleural cavity and fissures

**Fig. 13:**
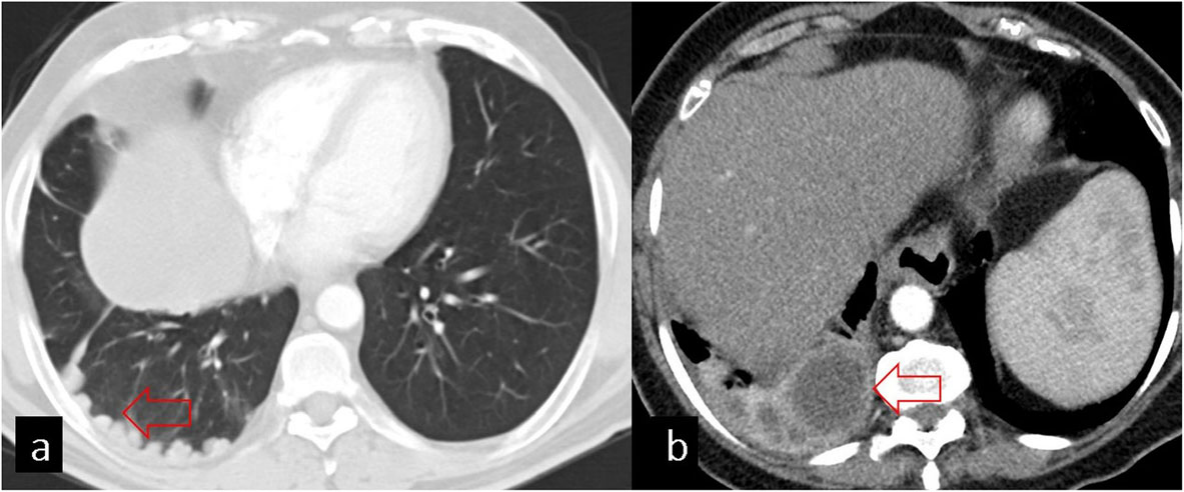
**a** Hydatid cysts of the pleura and fissure. **b** Right pleural cystic lesion with peripheral contrast enhancement that belongs to empyema

As stated above in respect to the rupture of pulmonary HC, HC can perforate into the pleural cavity and cause pleural effusions, hydropneumothorax, simple and tension pneumothorax, and empyema [14, 16]. Although pleural hydatid disease is usually secondary to lung involvement, it may occasionally be primary. HC can be seen as unilocular or multilocular cysts in the pleural cavity. The pleural layers are avascular, and HC may occur and grow in this area due to the permeability of the cyst membrane to calcium, chloride, potassium, water, and urea. Most HCs located in the pleural cavity are attached to the visceral pleura with a thin pedicle [25, 26]. HCs in the pleural cavity can be located in both the subpleural region and fissures. The differential diagnosis of pleural HC should include empyema, dermoid cyst in the pleural cavity, mesothelioma, synovial sarcoma, bronchogenic cyst, lymphoma, and pleural metastases (Fig. 13).

## Diaphragm

**Fig. 14:**
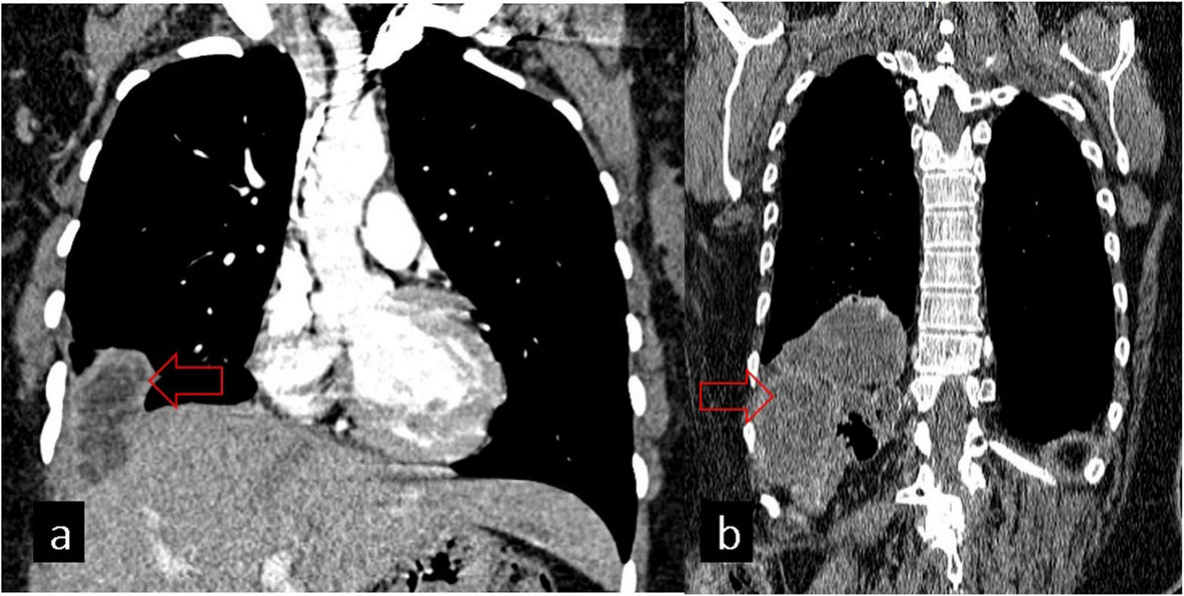
Chest CT images on the coronal plane show transdiaphragmatic migration of hydatid cysts in different patients (**a**, **b**, red arrows). Diaphragmatic discontinuation can be observed

Although it is not common for HCs to be located in the diaphragm, they may be seen as primary or secondary due to the extension of a hepatic or pulmonary HC. Transdiaphragmatic migration of HC from the posterior segments of the right liver lobe has been reported to be a more common complication because of the proximity to the diaphragm. Furthermore, the bare area of the liver is the most common way of transdiaphragmatic migration (Fig. 14). This is probably related to the lack of peritoneal coverage in this particular area and decreased resistance to cyst growth. Transdiaphragmatic migration can be seen as an hourglass-shaped lesion or loculated pleural effusion in the posterior thorax on the lateral projection. Although CT is very useful in demonstrating the location and extension of HC, it cannot easily show the diaphragmatic defect. MRI can better delineate the cyst structure and the diaphragmatic defect. The involvement of the diaphragm usually requires diaphragmatic repair after cyst removal [2, 26].

## Mediastinal findings

**Fig. 15:**
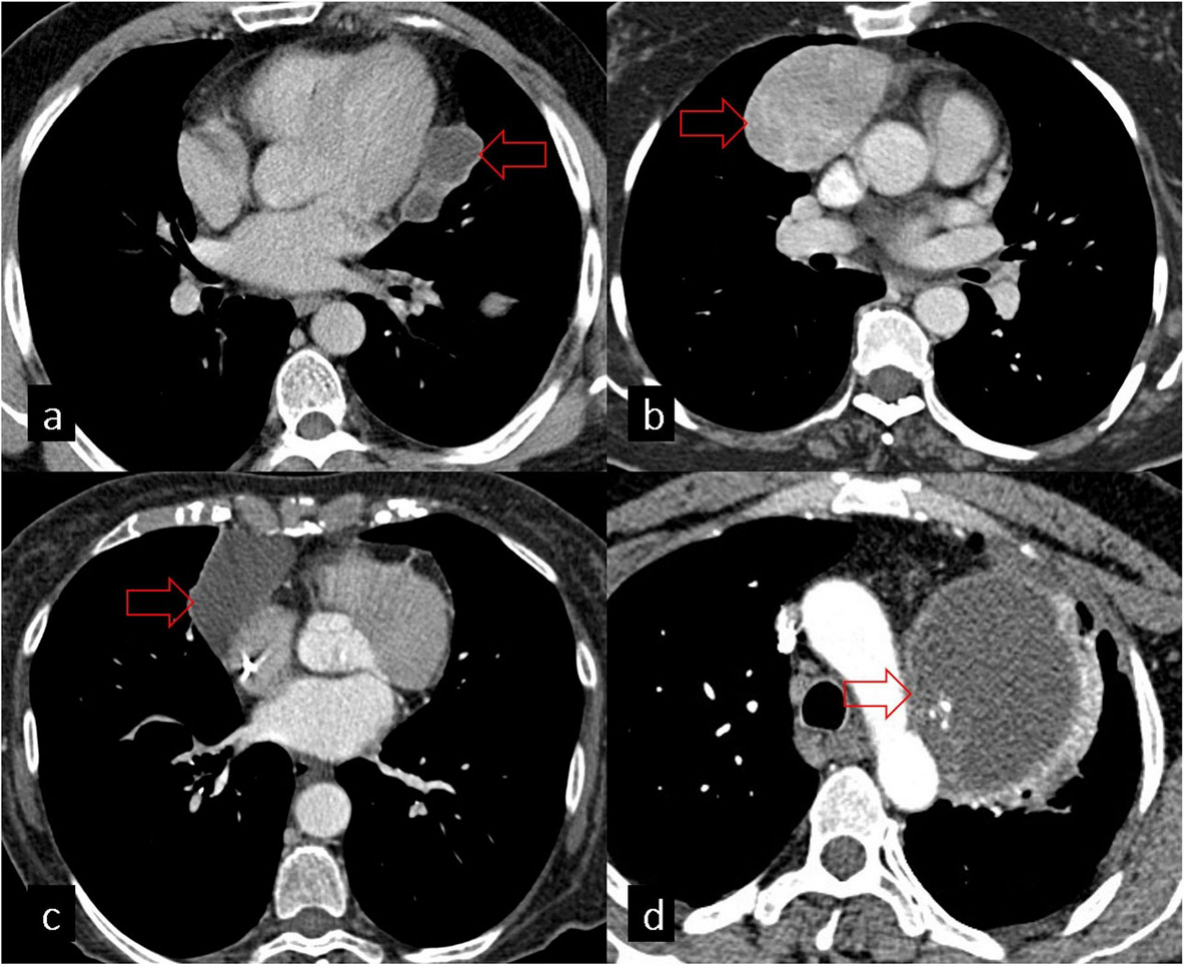
**a** Axial CT image shows a cystic lesion in the mediastinum, adjacent to the left ventricle, pathologically proven as a hydatid cyst (red arrow). **b**, **c**, **d** Differential diagnosis of mediastinal hydatid cysts: thymoma (**b**), pericardial cyst (**c**), and teratoma (**d**)

Mediastinal HCs are rare with an incidence ranging from 0 to 6%. They may be primary or secondary to spread from the lung, liver, or spleen [7]. It has been reported that approximately 36% of primary mediastinal HCs are in the thymic area of the anterior mediastinum [26]. The imaging appearance of mediastinal HCs can vary including unilocular, multilocular, complicated, and calcified cysts. Thymoma, teratoma, and pericardial cysts should also be considered in the differential diagnosis of HCs located in the anterior mediastinum [7] (Fig. 15).

In addition to anterior mediastinum localization, there can be HC involvement of the heart and vascular structures. Clinical symptoms depend on the size and involvement of adjacent structures.

### Cardiac involvement

**Fig. 16:**
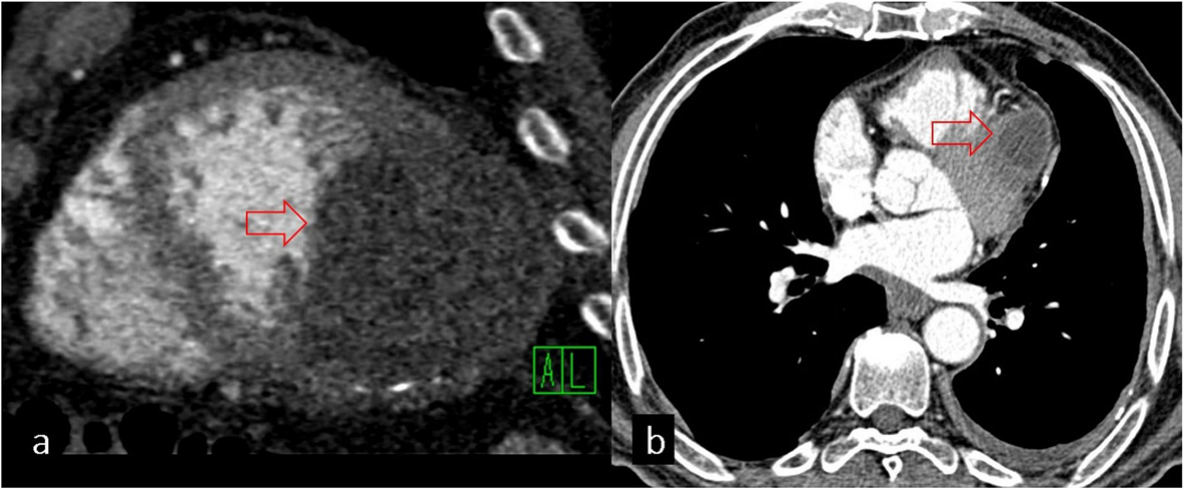
Cardiac involvement of hydatid cyst. **a** Hydatid cyst with calcification in the left ventricle is seen on the short-axis CT image (red arrow). **b** Hydatid cyst in the left ventricle is demonstrated in another patient (red arrow)

The heart is an unusual location for HC with an incidence of 0.02–2% and may occur because of hematogeneous spread or rupture of lung HC [27]. The most common locations of cardiac involvement (in decreasing order of frequency) are the left ventricle (50–60%), interventricular septum (10–20%), right ventricle (5–15%), pericardium (10–15%), and atriums (5–8%) [28]. Cardiac HCs can be diagnosed with transthoracic echocardiography, CT, and MRI. Transthoracic echocardiography can be limited in showing the relationship between the cyst and adjacent structures. CT is the best modality for demonstrating calcification of the cyst wall (Fig. 16) but maybe inadequate for the visualization of the heart chambers because of cardiac motion artifact. However, cardiac-gated MRI provides information about the internal structure of HCs, their extension to adjacent structures, and the effects of HCs on cardiac function [7].

Clinically, cardiac HC can be fatal, and symptoms can be seen due to the compression of large mediastinal cysts on vital organs such as the esophagus, trachea, or vascular structures [7]

### Involvement of vascular structures

**Fig. 17:**
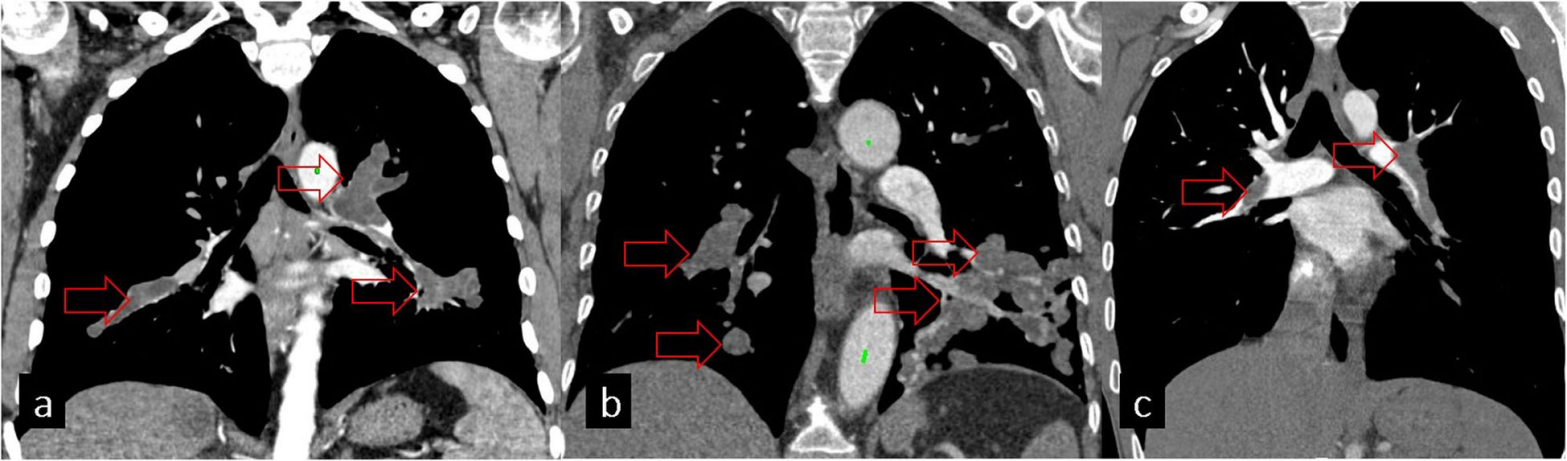
**a** Hydatid cysts of pulmonary arteries seen on the coronal chest CT image. **b** Sarcoma of the pulmonary artery shows vascular distension and fills the lumen like hydatid cysts of the pulmonary arteries. **c** Pulmonary thromboembolism as another differential diagnosis of hydatid cyst seen on the coronal chest CT image

Although the involvement of vascular structures is very unusual, both arteries and venous structures can be involved by HCs. The most frequent cause is the embolism of HC from primary cardiac locations. The inferior vena cava can also be involved by liver HCs, and in this way, HCs can spread to the right cardiac chambers [29, 30]. Widening of the vascular lumen due to rounded intravascular masses with levels of fluid attenuation can be seen on CT angiography. MR is more useful in the diagnosis of intravascular HCs with typical low signal intensity on T1-weighted and high signal intensity on T2-weighted images. Other reasons for intraluminal filling defects such as thromboembolism and vascular sarcomas should also be kept in mind in the differential diagnosis (Fig. 17). Patients with unruptured vascular HCs can be asymptomatic for years because of the slow growth pattern of the cyst. Symptoms including cough, hemoptysis, shortness of breath, and chest pain can develop when HCs enlarge, and rupture may lead to anaphylactic shock or even death.

**Fig. 18:**
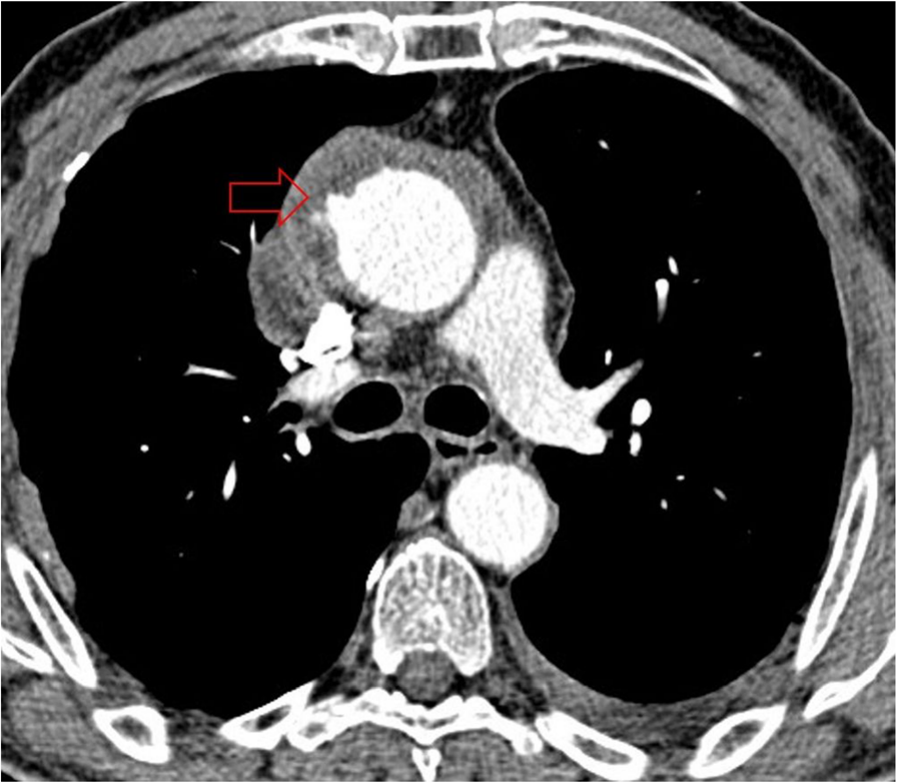
Axial chest CT image in the mediastinal window shows pseudoaneurysm formation and wall erosion in ascending aorta (red arrow)

The involvement of the thoracic aorta is exceptionally rare, but in such cases, pseudoaneurysm and wall erosion can be seen on CT images (Fig. 18).

## Conclusion

The lungs are the second most frequent location of hematogenous spread of HC in adults and probably the most common location in children. In addition to pulmonary HC, HC may develop in any extrapulmonary region including the chest wall, pleural cavity, fissures and diaphragm, mediastinum, heart, and vascular structures. As symptoms are non-specific, imaging plays a crucial role in the diagnosis of hydatid cyst in common pulmonary and uncommon extrapulmonary locations. Imaging can also show complications and extension of hydatid cyst. A good knowledge of imaging findings and differential diagnosis of hydatid cyst will enable early diagnosis and guide the therapeutic management.

## Data Availability

Data sharing is not applicable to this article as no datasets were generated or analyzed during the current study.

## Abbreviations

CT: Computed tomography
EG: *Echinococcus granulosus*
HC: Hydatid cyst
MRI: Magnetic resonance imaging
